# Three-Dimensional Porous Ti_3_C_2_T_x_-NiO Composite Electrodes with Enhanced Electrochemical Performance for Supercapacitors

**DOI:** 10.3390/ma12010188

**Published:** 2019-01-08

**Authors:** Kaicheng Zhang, Guobing Ying, Lu Liu, Fengchen Ma, Lin Su, Chen Zhang, Donghai Wu, Xiang Wang, Ying Zhou

**Affiliations:** 1 Department of Materials Science and Engineering, College of Mechanics and Materials, Hohai University, Nanjing 211100, China; zhangkc1995@163.com (K.Z.); liulu201709@163.com (L.L.); mafengchen0729@163.com (F.M.); sulin_2016@163.com (L.S.); zhangchh2015@126.com (C.Z.); 2 Key Laboratory of Integrated Regulation and Resources Development of Shallow Lakes of Ministry of Education, College of Environment, Hohai University, Nanjing 210098, China; wdh1018@hhu.edu.cn (D.W.); yingzhouhhu@126.com (Y.Z.); 3 Key Laboratory of Superlight Materials & Surface Technology (Harbin Engineering University), Ministry of Education, Harbin 150001, China; wangxiang@hrbeu.edu.cn

**Keywords:** MXene, porous Ti_3_C_2_T_x_-NiO composite, freeze-drying, electrode, supercapacitor

## Abstract

Ti_3_C_2_T_x_ and Ti_3_C_2_T_x_-NiO composites with three-dimensional (3D) porous networks were successfully fabricated via vacuum freeze-drying. The microstructure, absorption, and electrochemical properties of the developed composites were investigated. Nickel oxide (NiO) nanoparticles could be evenly distributed on the three-dimensional network of three-dimensional Ti_3_C_2_T_x_ using solution processing. When employed as electrochemical capacitor electrodes in 1 M environmentally friendly sodium sulfate, Na_2_SO_4_, solution, the three-dimensional porous Ti_3_C_2_T_x_-NiO composite electrodes exhibited considerable volume specific capacitance as compared to three-dimensional porous Ti_3_C_2_T_x_. The three-dimensional porous Ti_3_C_2_T_x_-NiO composite delivered a remarkable cycling performance with a capacitance retention of up to 114% over 2500 cycles. The growth trend of the capacitance with NiO content shows that nickel oxide plays a crucial role in the composite electrodes. These results present a roadmap for the development of convenient and economical supercapacitors in consideration with the possibilities of morphological control and the extensibility of the process.

## 1. Introduction

Since the first report of graphene by Novoselov and Geim in 2004, there has been a great interest in the two-dimensional (2D) materials [1]. 2D materials are widely used in wastewater treatment, supercapacitors, lithium ion batteries, and reinforcements in composite due to their unique properties such as good adsorption performance, unusual electrochemical performance, and unique mechanical properties [2,3,4,5,6,7,8,9]. The 2D transition metal carbides (MXenes) were discovered by Gogotsi and Barsoum in 2011 [10]. These novel materials are expressed as M_n+1_X_n_T_x_, where M is a transition metal such as Ti, Ta, or Nb, X represents C and/or N, T_x_ denotes surface functional groups (-OH or -F), and the values of n are 1, 2, or 3. Similar to graphene, these kind of materials have two-dimensional structures, large specific surface area, and good electrochemical performance, magnetic, and mechanical properties. Thus MXenes are emerging as promising candidates for various applications such as supercapacitors, catalysis, adsorption, hydrogen storage, new polymer reinforced matrix composites, and several other fields [11,12,13,14]. There are 20 different MXenes. Ti_3_C_2_T_x_ is the first to be discovered and by far the most studied MXene. This material has shown excellent performance as the electrode material of supercapacitors with the performance exceeding those of most previously reported materials [15,16].

A three-dimensional (3D) porous structure is a possible approach to achieve high power density for energy storage applications. The 3D structure materials built of graphene and other 2D materials have attracted a significant attention [17,18,19,20,21,22,23]. 3D porous Ti_3_C_2_T_x_ can be prepared by vacuum freeze-drying method using a solvent as a template [24,25]. As the 3D structure prevents the restacking of MXene nanosheets, a high electroadsorption capacity and promising potential for desalination applications have been reported [25]. Also, 3D porous MXene (Ti_3_C_2_T_x_)–rGO (reduced graphene oxide) composite aerogel electrode has been fabricated for microsupercapacitor applications, which can display an area specific capacitance of 34.6 mF cm^−2^ at a scan rate of 1 mV s^−1^ and a cycling performance with a capacitance retention up to 91% over 15,000 cycles [24]. However, there are still limited reports about the application in energy storage of 3D MXene and their development still remains a challenge.

Metal oxides such as NiO [26], RuO_2_ [27], Fe_3_O_4_ [28], Fe_2_O_3_ [29], and Co_3_O_4_ [30] are always the important materials for electrodes. Because the double-layer capacitance is due to physical adsorption and desorption processes while the pseudo-capacitance is associated with redox reactions, such as those observed in transition metal oxides or hydroxides and conductive polymers, the pseudo-capacitance produced by a faradaic reaction of the metal oxides at the electrode/solution interface is greater than the double-layer capacitance on the surface of the active material [31,32], these materials have broad research prospects and have attracted much attention from the researchers. As NiO is easily synthesized and has relatively high specific capacitance [33], Hong-zhi Yang et al. prepared Nickel oxide (NiO) hollow microspheres which exhibited a high specific capacitance of about 1340 F/g at a current density of 1 A/g [26]. As the production process is economical and eco-friendly, NiO is regarded as one of the most ideal super capacitor electrode materials [34].

In this work, we introduced metal oxide NiO into Ti_3_C_2_T_x_ to modify the energy storage of MXene. 3D porous Ti_3_C_2_T_x_-NiO composite electrodes were first successfully fabricated by vacuum freeze-drying method from the aqueous solutions of Ti_3_C_2_T_x_ and NiO. Ti_3_C_2_T_x_ and NiO were well combined and NiO was uniformly distributed on the 3D network skeleton. The effect of NiO content on the performance was thereafter discussed. We have demonstrated that 3D Ti_3_C_2_T_x_-NiO composite electrodes achieved a high capacitance performance and NiO played a vital role.

## 2. Experimental

### 2.1. Synthesis of Ti_3_AlC_2_

Before the production of Ti_3_C_2_T_x_, we first synthesized Ti_3_AlC_2_ powder via hot pressing sintering method. The latter was produced by mixing TiC (99 wt. %, 2–4 µm, Aladdin Industrial Co., Shanghai, China), Ti (99.9 wt. %, −45 μm, Jinzhou Institute of Metal Material, Jinzhou, China) and Al (99.7 wt. %, −29 μm, China Northeast Light Alloy Co., Harbin, China) powders according to the molar ratio of 1.8:1.2:1.1. The mixed powder (100 g) was pre-compacted in a steel die using a load corresponding to a stress of 30 MPa. The pre-compact was heated in a tube furnace under flowing Ar to 1350 °C at a heating rate of 10 °C/min and held at that temperature for 1h before furnace cooling. The resultant lightly sintered Ti_3_AlC_2_ was then milled with a drill machine (Z516, Yongkang dongcheng fenjin machinery factory, Yongkang, China) and passed through a −325 mesh sieve, which was used for further experiments.

### 2.2. Synthesis of Ti_3_C_2_T_x_ MXene

1 g LiF (99 wt. % purity, Shanghai Macklin Biochemical Co., Ltd., Shanghai, China) and 10 mL of 12 M HCl (AR, Shanghai Lingfeng Chemical Reagent Co., Shanghai, China) were mixed as the etching solution of Ti_3_AlC_2_ powder. Thereafter, 1 g Ti_3_AlC_2_ powder was immersed in the etching solution and stirred at 35 °C for 24 h. Then, the resultant mixture was washed with deionized water several times and centrifuged at a speed of 3500 rpm (3 min for each cycle) until the pH value of the supernatant was ≥ 6. Then, the multilayer Ti_3_C_2_T_x_ (PH ≥ 6) was sonicated for 1 h at room temperature under bubbling Ar to get the monolayer Ti_3_C_2_T_x_. Thereafter, the resultant exfoliated solution was centrifuged at a speed of 3500 rpm for 30 min. Finally, the supernatant suspension was an aqueous suspension of monolayer Ti_3_C_2_T_x_ and was stored under Ar for further experiments. The suspension concentration in this experiment was 13.6 mg/mL.

### 2.3. Preparation of 3D Porous Ti_3_C_2_T_x_-NiO Composites Electrodes

The prepared Ti_3_C_2_T_x_ suspension was slowly added to NiO (99.5 wt. %, < 30 nm, Aladdin Industrial Co., Shanghai, China) suspension which was dispersed in water firstly by magnetic stirrer for 30 min. The weight ratios of Ti_3_C_2_T_x_:NiO = 4:1, 2:1, 1:1, and 1:2 were considered in this work. Then, the suspension was stirred with a magnetic bar for 30 min and sonicated (120 W) for 10 min for better dispersion of NiO. After the mixing process, the suspension was poured into the mold and then pre-freezed at −80 °C for 24 h followed by vacuum freeze-drying at −50 °C for 2 days. A cylindrical plastic mold with a diameter of 7 cm and a height of 1 cm was used in this experiment. By cutting or tearing the prepared 3D porous Ti_3_C_2_T_x_-NiO composites into a size of about 5 × 5 mm^2^, the electrodes were prepared to facilitate electrochemical testing. The weight of the electrodes should be measured each time before electrochemical testing, the mass of the electrodes in this experiment were between 1 mg and 2 mg.

### 2.4. Material Characterization

The X-ray diffraction patterns of the samples were tested using an X-ray diffractometer (Rigaku Smartlab, Rigaku Corporation, Tokyo, Japan) with Cu (Kα) at a speed of 2°/min. The microstructures of the samples were observed under a Scanning Electron Microscope (SEM, S-4800, Hitachi Co., Tokyo, Japan). The densities of the fabricated samples were determined using the Archimedes method.

### 2.5. Electrochemical Performance Tests

The electrochemical performance of the 3D porous Ti_3_C_2_T_x_-NiO composites electrodes was measured with Cyclic Voltammetry (CV) and galvanostatic charge-discharge tests by using a CHI-660 electrochemical workstation (Chenhua Instruments Co., Shanghai, China). 1 M Na_2_SO_4_ aqueous solution was used as the electrolyte. All the electrochemical tests were conducted in a three-electrode system. The Ti_3_C_2_T_x_-NiO composites were used as the working electrodes and the Pt plate was selected as the counter electrode. CV scans were recorded from −0.8 V to −0.2 V (vs. Ag/AgCl) at the scanning rates from 2 mV/s to 100 mV/s. In the range of 10^−2^ Hz to 10^5^ Hz, the same three-electrode cell configuration described above was used for electrochemical impedance spectroscopy (EIS) at a potential amplitude of 5 mV. Galvanostatic cycling was carried out at a current density of 1 A/g.

## 3. Results and Discussion

**Figure 1:**
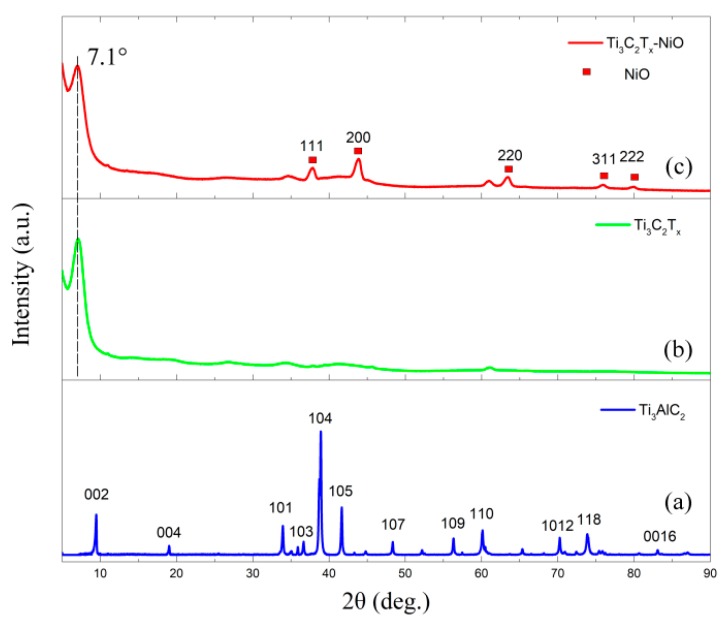
XRD patterns of (**a**) Ti_3_AlC_2_ produced by hot pressing, (**b**) pure Ti_3_C_2_T_x_ etched by LiF + HCl solution, and (**c**) 3D porous Ti_3_C_2_T_x_-NiO composite.

The XRD patterns of as-prepared Ti_3_AlC_2_ powders, 3D porous Ti_3_C_2_T_x_, and Ti_3_C_2_T_x_-NiO composite are shown in Figure 1. Typical X-ray diffractometer (XRD) pattern of Ti_3_AlC_2_ indicates that the precursor of Ti_3_C_2_T_x_ was pure, as shown in Figure 1a. As shown in Figure 1b, a very sharp scattering peak of Ti_3_C_2_Tx appeared at 2θ = 7.1° for (002) for 3D porous Ti_3_C_2_T_x_ after etching by LiF + HCl solution and freeze-drying method. No other oxide peaks could be observed. The *c*-lattice parameters, *c*-LP, of 3D porous Ti_3_C_2_T_x_—24.88 Å were comparable to the reported values [35]. NiO addition had no effect on the *c*-LP of 3D porous Ti_3_C_2_T_x_ as shown in Figure 1c, where typical Ti_3_C_2_T_x_ and NiO peaks could be found in the 3D porous Ti_3_C_2_T_x_-NiO composites.

**Figure 2:**
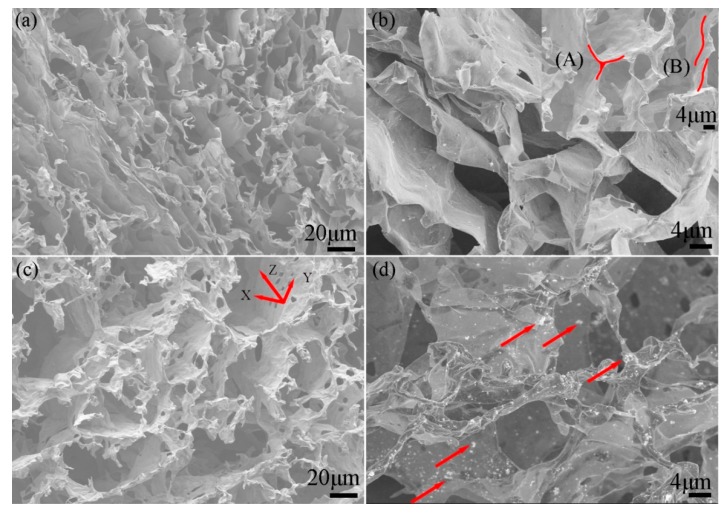
SEM images of the (**a**) 3D porous Ti_3_C_2_T_x_, (**b**) skeleton construction, (**c**) pores in the wall, and (**d**) 3D porous Ti_3_C_2_T_x_-NiO composite with weight ratios of 2:1.

To further evaluate the hollow structure of the 3D porous Ti_3_C_2_T_x_ and the dispersion of NiO in the composite electrodes, the samples were examined under SEM (see Figure 2 and Figure S2). SEM images confirmed that the porous 3D Ti_3_C_2_T_x_ had interconnected frameworks with an open macroporous structure (Figure 2a,b). The porous 3D Ti_3_C_2_T_x_ was clearly full of holes and most of the monolayer Ti_3_C_2_T_x_ was arranged in the direction of water crystallization after freeze-drying. 2D Ti_3_C_2_T_x_ overlapped with each other to form a 3D network (see Figure 2b). The monolayer Ti_3_C_2_T_x_ could overlap like a three-dimensional skeleton or it could overlap on a plane (see the red curves in the inset of Figure 2b). The various overlapping methods successfully provided Ti_3_C_2_T_x_ with a three-dimensional structure, like a porous foam. Figure 2c illustrates that in addition to the holes in the parallel direction, there were also pores in the vertical direction due to incomplete overlap between Ti_3_C_2_T_x_ layers. The 3D network framework constructed by 2D Ti_3_C_2_T_x_ made NiO (the red arrows in Figure 2d) distributed on its surface.

The porous structure provided the composite an excellent absorption capacity for deionized water, as shown in Figure S1a,b. As MXenes are extremely hydrophilic, the cylindrical 3D Ti_3_C_2_T_x_ quickly absorbed large amounts of water in a few seconds. 3D porous Ti_3_C_2_T_x_ had a specific surface area which was tested to 230 m^2^/g. The density of the 3D porous Ti_3_C_2_T_x_ and 3D porous Ti_3_C_2_T_x_-NiO composites with different initial weight ratios of 4:1, 2:1, 1:1, and 1:2 of Ti_3_C_2_T_x_-NiO were measured as 8.6 ± 0.2, 10.8 ± 0.2, 12.9 ± 0.2, 17.2 ± 0.1, and 25.8 ± 0.1 mg/cm^3^ and the 3D Ti_3_C_2_T_x_ absorbed 22 times its weight in deionized water with pink ink. Other researchers have reported the absorption of porous 2D materials as graphene. For example, Ruiyang Zhang et al. prepared g-C_3_N_4_/graphene oxide wrapped sponge with an absorption capacity of 49.8 g/g for n-hexane [22]. Chao Gao et al. found that ultra-flyweight aerogels (UFA) exhibit a high absorption capacity of 290 g/g for crude oils [17]. Graphene sponge made by Wencai Ren et al. could absorb 129 times oil of its weight [23], while Graphene-based sponge made by Duc Dung Tai et al. demonstrated absorption capacities of 1.12 ton/m^3^ for soybean oil and 1.86 ton/m^3^ for chloroform, respectively [20]. As the density of Ti_3_C_2_T_x_ is several times greater than that of graphene, the absorption capacity of 3D porous Ti_3_C_2_T_x_ was relatively better. The absorption capacities obtained for 3D porous Ti_3_C_2_T_x_ and its composites in this work were compared to the values reported in literature and are summarized in Table 1.

**Figure 3:**
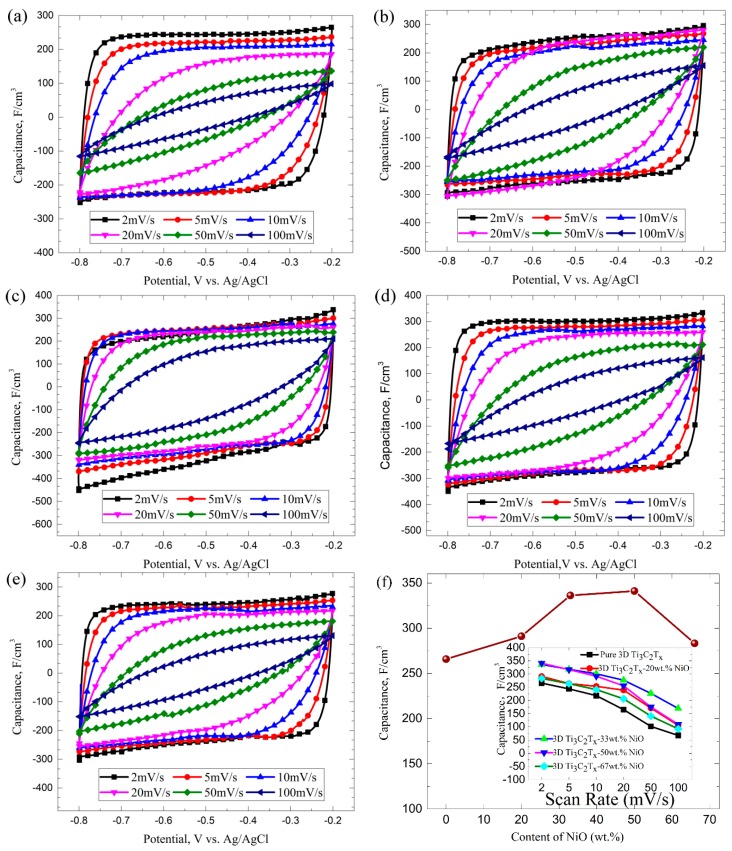
Electrochemical performance of 3D porous Ti_3_C_2_T_x_ and Ti_3_C_2_T_x_-NiO composites. Cyclic voltammetry data collected at scan rates from 2 to 100 mV/s for (**a**) pure 3D porous Ti_3_C_2_T_x_, (**b**) 3D porous Ti_3_C_2_T_x_-20 wt. % NiO, (**c**) 3D porous Ti_3_C_2_T_x_-33 wt. % NiO, (**d**) 3D porous Ti_3_C_2_T_x_-50 wt. % NiO, and (**e**) 3D porous Ti_3_C_2_T_x_-66 wt. % NiO. (**f**) The NiO content and scan rate dependence of specific capacitance.

Considering the unique porous microstructure of 3D Ti_3_C_2_T_x_ and NiO composite, we further evaluated its electrochemical performance as electrode in a three-electrode system (see Figure 3 and Figure S3). Figure 3a–e shows the cyclic voltammogram (CV) curves at all sweeping rates of pure 3D porous Ti_3_C_2_T_x_ and 3D porous Ti_3_C_2_T_x_-NiO composite electrodes produced by freeze-drying method with different initial weight ratios of 4:1, 2:1, 1:1, and 1:2 of Ti_3_C_2_T_x_-NiO. The capacitance was reduced due to incomplete Na^+^ and SO_4_^2−^ ion diffusion in the electrolyte. When the scanning speed reached 100 mV/s, the curves had some distortion and did not look like a rectangle. The area of the capacitance curve gradually decreased as the scanning rate increased, which is due to the slower transmission speed of ions in the electrolyte relative to the scanning rate (see Figure 3f). These results are consistent with the previous reports on conductive supercapacitors [24,25,36]. Figure 3f also illustrates that the specific capacitance obtained at 2 mV/s is about 341 F/cm^3^ with a 1:1 initial weight ratio. This value is much higher than those for the other samples. The results of these parallel experiments on different initial weight ratios strongly indicate the importance of NiO to improve the electrochemical performance. The nickel oxide in 3D porous Ti_3_C_2_T_x_-NiO composite electrodes provided an excellent faraday capacity at the time of testing. According to the report that only electric double layer capacitance was recognized in the in neutral electrolytes [37], the excellent faraday capacity of NiO could be produced not only on the surface of the electrode but also in the whole electrode and it was better than the capacitance of Ti_3_C_2_T_x_ itself; hence, higher capacitance and energy density could be obtained. When under the same electrode area, the pseudocapacitor can be 10–100 times the capacity of the double layer [30,38]. Moreover, we compared our results with major published data on porous 2D materials and have listed the major characteristics of each report, such as test configuration, specific capacitance, capacitance retention, and electrolyte in Table 2.

**Figure 4:**
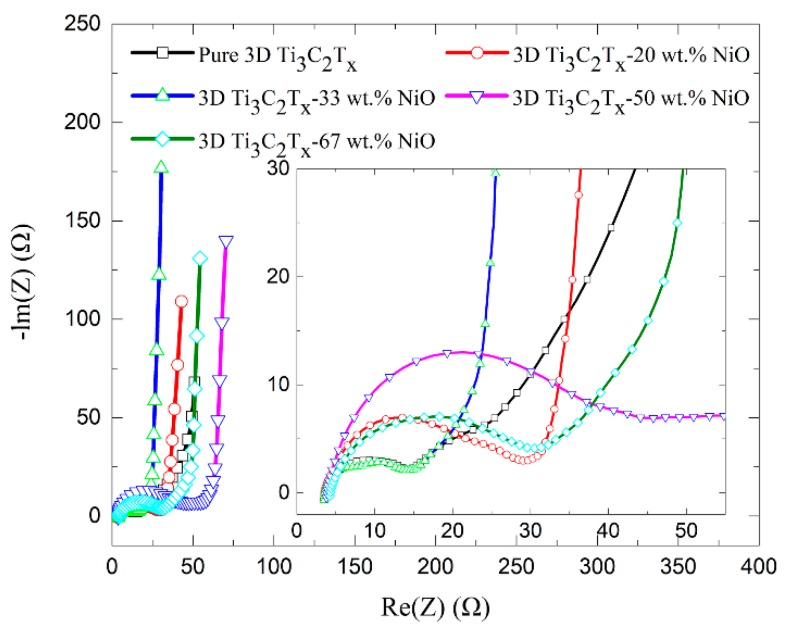
Nyquist plots of pure 3D porous Ti_3_C_2_T_x_ and 3D porous Ti_3_C_2_T_x_-NiO composite.

To investigate the ion transport behavior and resistance of the electrode of the samples, electrochemical impedance spectroscopy (EIS) was conducted and the results were shown in Figure 4. Electrochemical impedance spectroscopy could be divided into two regions called knee frequencies, the high frequency region being a semicircular arc and the low frequency region being a straight line. The diameter of the semicircle in the high-frequency range determines the value of the charge transfer resistance (Rct), which was derived from the ion transfer across the interface of the Ti_3_C_2_T_x_ wafer in contact with the electrolyte solution. Capacitive impedance appeared in the wide frequency range and the fast increase of the imaginary part of the low frequency impedance illustrates a capacitance storage mechanism. The low frequencies regions (the straight lines) reflected the Warburg resistance. These results were consistent with the previous reports on Ti_3_C_2_T_x_ membrane material or conducting Ti_3_C_2_T_x_ “clay” electrolytes [37,39]. Although the forms and structures of the electrodes are different, comparable capacitive impedance over a wide frequency range shows the intrinsic properties of the same active material in Ti_3_C_2_T_x_ and this property is not altered with the addition of nickel oxide.

**Figure 5:**
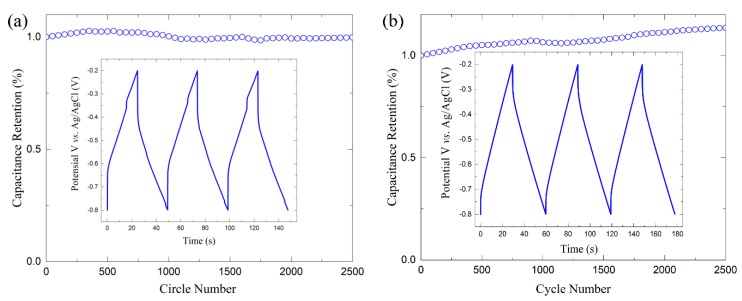
Cycling performance tested by galvanostatic charge-discharge conducted at 1 A/g for (**a**) pure 3D porous Ti_3_C_2_T_x_ and (**b**) Ti_3_C_2_T_x_-50 wt. % NiO.

In order to explore the cyclability of the prepared electrodes, pure 3D porous Ti_3_C_2_T_x_ and 3D porous Ti_3_C_2_T_x_-NiO were also evaluated and results are presented in Figure 5a,b, respectively. Cycling performance was tested by galvanostatic charge-discharge testing at a current density of 1 A/g. The nontriangular shapes of the voltage vs. time profiles shown in the insets are consistent with the pseudocapacitive nature of the charge storage mechanism suggested by the corresponding CVs. The coulombic efficiency of the pure 3D porous Ti_3_C_2_T_x_ and 3D porous Ti_3_C_2_T_x_-NiO were 99% and 103%, respectively. A slight increase of ~3% of the initial capacitance could be observed for the pure 3D porous Ti_3_C_2_T_x_ electrode after ~1000 cycles, which may be ascribed to the intercalation of Na^+^ into the interlayer space of Ti_3_C_2_T_x_, leading to an enhanced capacitance [25]. The capacitance retention was as high as 100% over 2500 cycles, as presented in Figure 5. No capacitance loss was observed for 3D porous Ti_3_C_2_T_x_-50 wt. % NiO electrode after 2500 cycles (Figure 5b). An increase of ~14% of the initial capacitance could be observed for the pure Ti_3_C_2_T_x_-50 wt. % NiO electrode after 2500 cycles. The cyclic stability of metal oxides is relatively poor due to chemical reactions [40,41,42], but 3D porous Ti_3_C_2_T_x_ framework itself has good cyclic stability and improve the ion accessibility during the cycling process, which leads to an increased accommodation behavior for charges [36]. The specific capacitance and cyclic stability obtained for 3D porous Ti_3_C_2_T_x_ and its composite in this work were compared to the values reported in the literature and summarized in Table 2.

## 4. Conclusions

We produced pure 3D porous Ti_3_C_2_T_x_ and 3D porous Ti_3_C_2_T_x_-NiO composite electrodes with different initial weight ratios by freeze-drying method. As per the absorption experiments in water and oil, the 3D porous Ti_3_C_2_T_x_ had a remarkable and comparable absorption capacity. The electrochemical performance of pure 3D porous Ti_3_C_2_T_x_ and 3D porous Ti_3_C_2_T_x_-NiO composite electrodes was studied in the scan rate range of 2–100 mV/s using 1 M Na_2_SO_4_ as the electrolyte. The composite electrodes effectively combined the advantages of Ti_3_C_2_T_x_ and nickel oxide. The addition of nickel oxide improved the specific capacitance of the composite electrode and the 3D porous Ti_3_C_2_T_x_ framework ensured relatively good cyclic stability of the composite electrodes. The volumetric specific capacitance of the electrode was maximum for the initial weight ratio of 1:1 for Ti_3_C_2_T_x_-NiO. Adding NiO was an effective way to improve the specific capacitance of 3D MXene. The capacitance retention of the 3D porous Ti_3_C_2_T_x_-NiO composite electrodes was superior to the analogous materials reported in several literatures.

## Figures and Tables

**Table 1:** Summary of absorption capacities of formulated 3D porous Ti_3_C_2_T_x_ compared to the values reported in other literature.

Materials	Solvent	Absorption Capacity		Reference
		Ms/M_M_ (g/g)	Ms/V_M_ (g/cm^3^)	
porous 3D Ti_3_C_2_Tx	deionized water	22	72.6	This work
	machine oils	14.6	48.2	
g-C_3_N_4_/GO-wrapped sponge	n-hexane	49.8	-	[22]
UFA	crude oils	290	-	[17]
graphene sponge	oils	129	-	[23]
graphene-based sponge	soybean oil	-	1.12	[20]
	chloroform	-	1.86	

**Table 2:** Summary of specific capacitance and cyclic stability obtained for formulated porous 3D Ti_3_C_2_Tx and Ti_3_C_2_T_x_-NiO compared to the values reported in other literature.

Materials	Specific Capacitance	Capacitance Retention (%)	Electrolyte	Refs.
porous 3D Ti_3_C_2_T_x_	266 F/cm^3^, 67 F/g 2 mV/s	100% over 2500 cycles	1 M Na_2_SO_4_	This work
porous 3D Ti_3_C_2_T_x_-20 wt. % NiO	291 F/cm^3^, 72 F/g 2 mv/s	-	1 M Na_2_SO_4_	
porous 3D Ti_3_C_2_T_x_-33 wt. % NiO	336 F/cm^3^, 85 F/g 2 mv/s	-	1 M Na_2_SO_4_	
porous 3D Ti_3_C_2_T_x_-50 wt. % NiO	341 F/cm^3^, 77 F/g 2 mv/s	114% over 2500 cycles	1 M Na_2_SO_4_	
porous 3D Ti_3_C_2_T_x_-67 wt. % NiO	283 F/cm^3^, 56 F/g 2 mv/s	-	1 M Na_2_SO_4_	
Graphene oxide and resol aerogel	99 F/g ^a^, 100 mA/g	97% over 10,000 cycles	6 M KOH	[21]
rGO aerogel/NF	366 F/g, 2 A/g	60% over 1000 cycles	6 M KOH	[19]
3D GA-based mesoporous carbon	226 F/g, 1 mv/s	142% over 5000 cycles	1 M H_2_SO_4_	[36]
porous carbon nanofibers	202 F/g, 1 A/g	97% over 3000 cycles	6 M KOH	[18]
porous MXene	410 F/cm^3^, 5 mv/s	103% over5000 cycles	1 M NaCl	[25]
3D MXene-rGO aerogel	34.6 mF/cm^2 a^, 1 mv/s	91% over 15,000 cycles	-	[24]

^a^: two-electrode configuration.

## Supplementary Materials

The following are available online at http://www.mdpi.com/1996-1944/12/1/188/s1.
