# Supplementary material for: Three-Dimensional Porous Ti3C2Tx-NiO Composite Electrodes with Enhanced Electrochemical Performance for Supercapacitors
Source: Materials (Basel). 2019 Jan 8;12(1):188. doi: 10.3390/ma12010188 (PMC6337570; doi:10.3390/ma12010188)
Supplement: Supplementary file 1 [file materials-12-00188-s001.pdf]

Supplementary Materials for

Three-dimensional porous $Ti_3C_2T_x$-NiO composite electrodes with enhanced electrochemical performance for supercapacitors

Kaicheng Zhang [1], Guobing Ying [1,*], Lu Liu [1], Fengchen Ma [1], Lin Su [1], Chen Zhang [1], Donghai Wu [3], Xiang Wang [2], Ying Zhou [3]

[1] Department of Materials Science and Engineering, College of Mechanics and Materials, Hohai University, Nanjing 211100, China

[2] Key Laboratory of Superlight Materials & Surface Technology (Harbin Engineering University), Ministry of Education, Harbin 150001, China

[3] Key Laboratory of Integrated Regulation and Resources Development of Shallow Lakes of Ministry of Education, College of Environment, Hohai University, Nanjing 210098, P.R.China

* Correspondence: yinggb2010@126.com, yinggb001@hhu.edu.cn; Tel.: +86-25-83787027; Fax: +86-25-83786046

Academic Editor: name

Received: date; Accepted: date; Published: date

Supporting Information

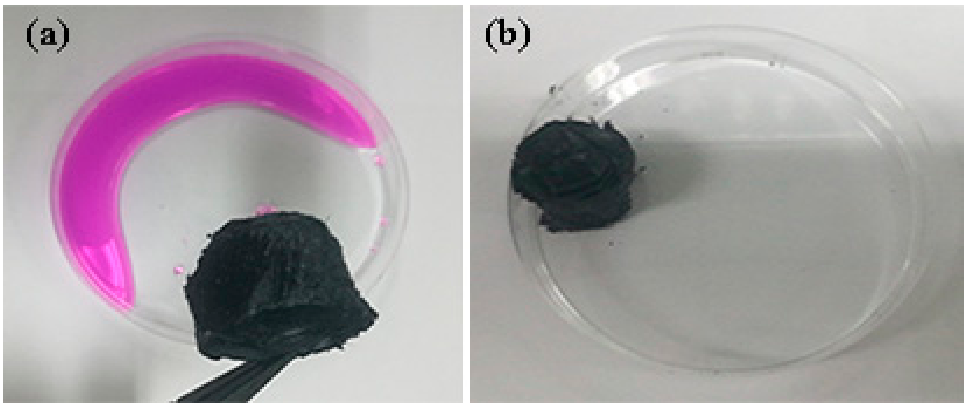

Fig. S1. The pictures for 3D porous $Ti_3C_2T_x$ (**a**) before and (**b**) after absorbing water.

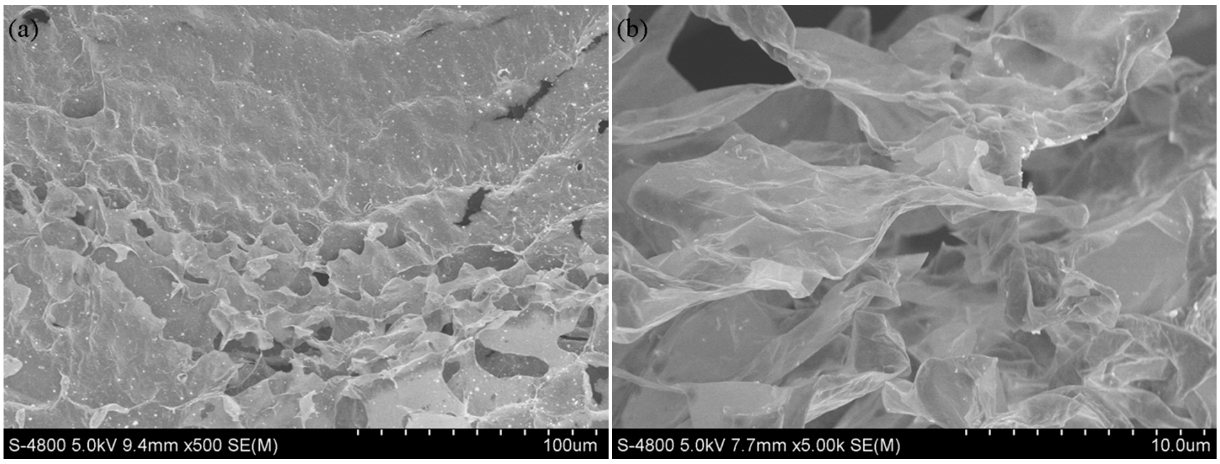

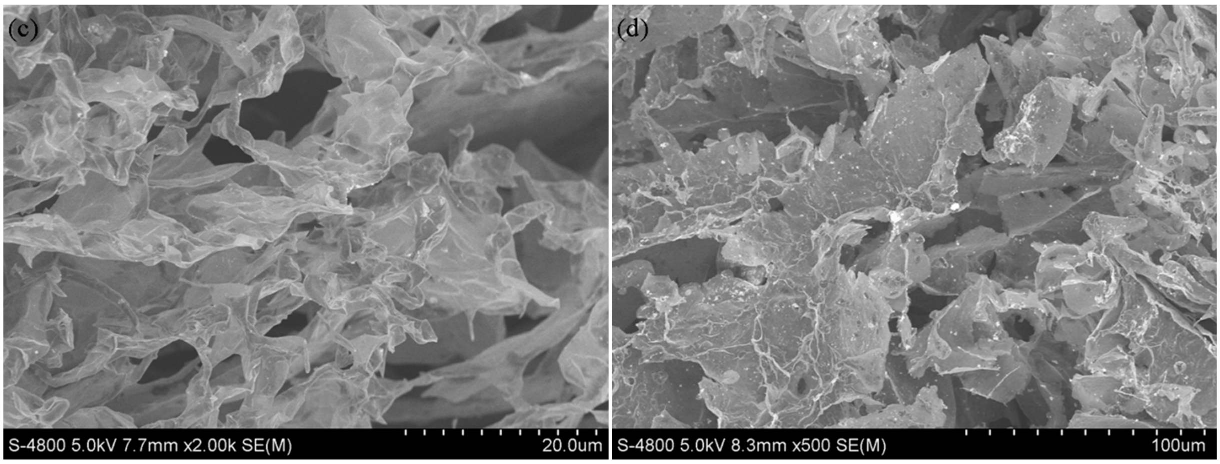

Fig. S2. SEM images of the (**a**) 3D porous $Ti_3C_2T_x$-NiO with initial ratio of 2:1, (**b**) 3D porous $Ti_3C_2T_x$-NiO with initial ratio of 1:1, (**c**) 3D porous $Ti_3C_2T_x$-NiO with initial ratio of 1:1, (**d**) 3D porous $Ti_3C_2T_x$-NiO with initial ratio of 1:2.

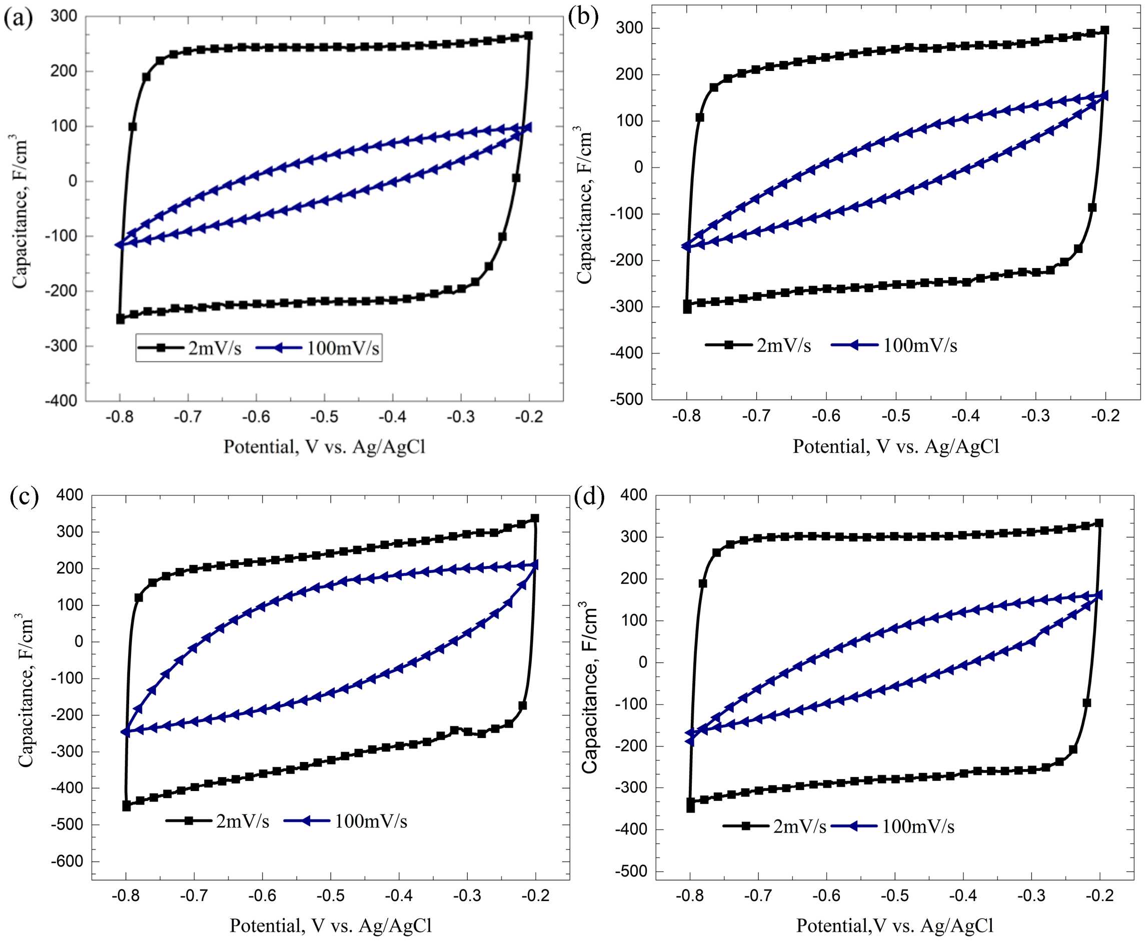

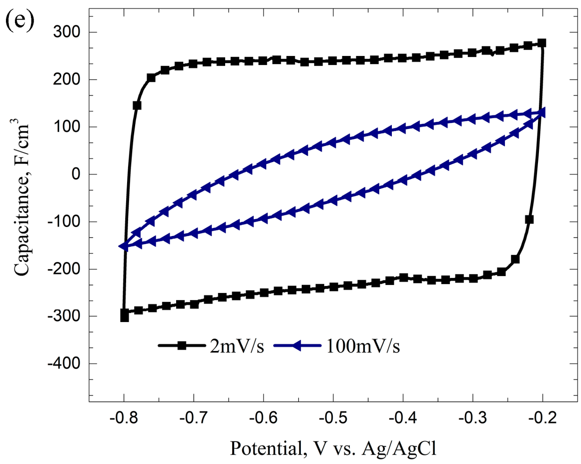

Fig. S3. CVs images of the (**a**) 3D porous $Ti_3C_2T_x$ (**b**) 3D porous $Ti_3C_2T_x$-NiO with initial ratio of 4:1, (**c**) 3D porous $Ti_3C_2T_x$-NiO with initial ratio of 2:1, (**d**) 3D porous $Ti_3C_2T_x$-NiO with initial ratio of 1:1, (**e**) 3D porous $Ti_3C_2T_x$-NiO with initial ratio of 1:2.
